# Delusion of Misidentifying of Parents as Infants as a Subtype of Intermetamorphosis Syndrome: A Case Report

**DOI:** 10.1155/crps/7415364

**Published:** 2025-05-26

**Authors:** Hirofumi Hirakawa, Takeshi Terao

**Affiliations:** Department of Neuropsychiatry, Oita University Faculty of Medicine, Yufu, Oita, Japan

## Abstract

Delusional misidentification syndromes (DMSs) are a group of disorders, characterized by consistent misidentification of individuals, locations, objects, or events. Four primary subtypes are recognized within this classification: Capgras syndrome, Frégoli syndrome, intermetamorphosis syndrome, and the syndrome of subjective doubles. We report a case of a woman with schizophrenia who experienced a strange delusion that her parents were her babies. This delusion did not arise from visual hallucinations of infants or from prosopagnosia. Furthermore, she denied experiencing auditory hallucinations related to the infants, suggesting that this delusion was not secondary to auditory hallucinations. The delusion that she had her infant was the delusion of maternity, and it was a form of delusional procreational syndrome that consists of sequential delusions in every possible stage of procreation. We concluded the delusion of misidentification that her parents were her own babies exhibited in this case was a subtype of intermetamorphosis syndrome coexisted with delusional procreation syndrome and her coping mechanisms shaped by underlying fears and inner wishes.

## 1. Introduction

Delusional misidentification syndromes (DMSs) are a group of disorders, characterized by consistent misidentification of individuals, locations, objects, or events [1]. Traditionally, four primary subtypes are recognized within this classification: Capgras syndrome, Frégoli syndrome, intermetamorphosis syndrome, and the syndrome of subjective doubles [2]. Capgras syndrome involves a delusional denial of recognizing familiar individuals, wherein the patients believe these individuals have been replaced by physically identical doubles who, however, lack the psychological attributes of the original persons [3]. In Frégoli syndrome, the patient maintains that a familiar person differs in physical appearance from the stranger but is psychologically the same person [4]. Intermetamorphosis syndrome represents a subtype in which the patient believes that the familiar person and the stranger have not only psychological but also physical similarities and that the misidentified individuals interchange with each other [5]. The syndrome of subjective doubles is characterized by the patient's delusional conviction that other individuals undergo a physical transformation, taking on the patient's own identity [6]. DMSs are often associated with schizophrenia, depression, bipolar disorders, and organic brain disorder [7, 8]. Although reliable data are not available, the prevalence of the DMSs in psychiatric patients ranges from 0.77% to 4%, and majority of the patients with DMSs were diagnosed with schizophrenia [9, 10]. Here, we report a case of a woman with schizophrenia who experienced a strange DMSs that her parents were her own babies.

## 2. Case Presentation

A 46-year-old Japanese woman with schizophrenia visited our hospital regularly. She was born as an only child, unmarried, and had never undergone pregnancy or childbirth. She was diagnosed with schizophrenia at the age of 25. As she was fond of children, she had once hoped to get married and have a child of her own; however, she abandoned that aspiration after being diagnosed with schizophrenia. She was primarily treated with a monthly dose of 100-mg long-acting injectable paliperidone and a daily dose of 1 mg brexpiprazole. She was occasionally experiencing auditory hallucinations and paranoid delusions. She lived calmly with her parents as a family of three but had minimal social interactions outside of them. As her parents were progressing in age—her father was 83-year-old, and her mother was 78-year-old—she feared their inevitable mortality and wished for an eternal life. She became suddenly convinced of and claimed that her parents were her newborn babies. She denied having any recollection of experiencing pregnancy or delivering before this strange delusion emerged. Instead, she believed, “God gave me these babies as a gift.” Although her religion was Buddhism, she was not particularly devout. She acknowledged that her parent's physical appearance remained unchanged and were in accordance with their respective ages and denied that their appearance was that of infants. She reported that her parents remained themselves in appearance and identity, while at other times, although their external appearance remained unchanged, their internal identities were switched to that of her babies. These changes occurred several times per week and sometimes even within a single day. She stated that her babies and her parents did not coexist simultaneously within her parents' bodies; rather, the identities alternated. She was uncertain about where her parents went when their identities changed to the babies, but she felt that their inner selves were clearly replaced. Regarding the babies, she explained that they were not her parents rejuvenated into infants (i.e., her parents in their childhood forms) but rather distinct individuals—her own babies. When her parents were not in the state of being her babies, they behaved as her ordinary father and mother. In the state in which her parents had become her babies, she stroked their heads as if soothing her own infants and called them “my sweet babies.” She firmly believed that her parents were her own newborn infants, although she was unable to explain the reason behind this conviction. She could only state that the belief arose from deep within her mind and that she simply felt it to be true. She denied experiencing any auditory hallucinations related to the sounds of crying infants or voices proclaiming that her parents were her infants. She demonstrated the ability to recognize the face of individuals indicating the absence of prosopagnosia. Aside from her parents, she did not perceive anyone in her close relationships as her own baby. This strange delusion lasted for about 3 months. On a particular outpatient visit, she was confused as her parents were her infants, and she cried and screamed. Consequently, a 25 mg intramuscular dose of levomepromazine was administered, which alleviated her agitated state, allowing her to return home. In the subsequent outpatient visits, she no longer complained that her parents were her babies, and her delusion improved.

## 3. Discussion

This case highlights a unique delusion in which the patient misidentified her parents and was convinced that they were her newborn infants. This delusion did not arise from visual hallucinations, pseudohallucinations, and imagery of infants or from prosopagnosia (i.e., selective visual agnosia characterized by the inability to recognize the identity of faces), as she affirmed that her parent's physical appearances remained unchanged. Furthermore, she denied experiencing auditory hallucinations related to the infants, suggesting that this delusion was not secondary to auditory hallucinations. The delusion that she had her infant was the delusion of maternity, and it was a form of delusional procreational syndrome that consists of sequential delusions in every possible stage of procreation, such as having a spouse, getting pregnant, giving birth, and assuming parental roles [11]. However, to the best of our knowledge, there are no reported cases of delusional procreational syndrome where the patient regards their own parents as their infants. DMSs are frequently comorbid with schizophrenia [12]. As the specific disintegration of subjectivity—often referred to as a self-disorder—is a notable psychopathological feature of schizophrenia, confusion about identity may occur under this condition. We discuss how this intriguing delusional belief may have emerged and developed in our patient.

Symptoms of DMSs are closely related to the environment, culture, or character of the patient; for example, a religious person believes in the transformation of people from patient's environment [7]. The false identification of a familiar person occurred, with the mother being reported as the most object of impaired identification [12]. Psychodynamic approaches may be useful for us to understand individual cases that DMSs result from ambivalent feelings, which are resolved by directing hate feelings onto an imagined double in order to retain the original intact [12]. Psychodynamic approaches also postulate that the various symptoms of schizophrenia have symbolic meaning for individual patients. Delusion may be substitutes for patient's inability to deal with objective reality and may be regressive, restitutive attemps to create a new reality or to represent inner wishes or fears.

We suggest the possible explanations for the origin of the patient's intriguing delusions: First, she could not explain the reason why she was convinced her parents were her infants in words and could only state that the belief arose from deep within her mind, which suggested this delusion emerged abruptly as a primary delusional idea. In this instance, the development of delusion was primary without explanation. Second, she lived with her parents and had minimal social interaction outside of her family; her parents constituted her entire social world. The approaching death of her parents also signified the collapse of her entire world. A secondary delusion that her parents were her own babies may have emerged as a coping mechanism to alleviate her fear of her parents' mortality and her inner wishes to preserve her parents and her world. Also, she used the interesting expression “God gave me these babies as a gift.” Her awareness of her parents' mortality underscored for her the reality that at her age she would never have infants and a family of her own, thus prompting her to imagine that her parents were the infants she would never have. She might have imagined her parents as her infants rather than imagining having infants of her own fathered by someone outside her nuclear family because of the extremely limited extent of her social network and interpersonal experience, residing as she did with her parents. In this context, the “gift” idea became a way for the patient to claim that she had infants without having given birth herself.

Finally, we discuss the type of DMS observed in this case. The patient complained a delusion in which her parents had internally transformed into her own baby despite maintaining their external appearance. Unlike Frégoli syndrome, in which the physical appearance of a familiar person changes, the subject in this case perceives the parent's appearance as unchanged. Furthermore, this case differed from the syndrome of subjective doubles in that the parents or her babies were not perceived as a double of the self. Therefore, we consider whether the misidentification in this case should be understood as Capgras syndrome or intermetamorphosis syndrome. Capgras syndrome is characterized by the delusion that a familiar person has been replaced by an identical-looking impostor. While our patient reported that outward appearance remained unchanged, her focus was on the belief that the internal identity of her parents had been transformed into that of her babies. Therefore, this case differed slightly from the notion that the parent had been replaced by an identical impostor. Intermetamorphosis syndrome is typically a brief that a familiar person and a stranger have interchange with each other both their physical appearances and identities [5]. In addition, intermetamorphosis syndrome also described that characterized by delusions that people have swapped identities while maintaining the same appearance, so it is not just disguise but a total transformation [13]. In this case, the “strangers” were her babies whom the patient had never seen before and who did not actually exist, meaning that there was no real physical form of her infants. Therefore, rather than imagining a fictional appearance for the babies, it is possible that the babies were expressed using the physical appearance of her parents who symbolically represents everything in her world. This interpretation may align with her delusion that the identities of her parents and the babies had swapped at times. From this perspective, this case may be understood as a subtype of intermetamorphosis syndrome. It is plausible that intermetamorphosis syndrome coexisted with delusional procreation syndrome and that her coping mechanisms, shaped by underlying fears and inner wishes, had contributed to the development of the unusual and intriguing belief that her parents had transformed into her own babies.

## 4. Conclusion

We concluded the delusion of misidentification that her parents were her own babies exhibited in this case was a subtype of intermetamorphosis syndrome. Further research and case reports are required to deepen the understanding of DMSs, which continues to captivate clinicians.

## Data Availability

The data that support the findings of this study are available from the corresponding author upon reasonable request.

## References

[1] Feinberg T. E., Roane D. M. (2005). Delusional Misidentification. *Psychiatric Clinics of North America*.

[2] Christodoulou G. N., Margariti M., Kontaxakis V. P., Christodoulou N. G. (2009). The Delusional Misidentification Syndromes: Strange, Fascinating, and Instructive. *Current Psychiatry Reports*.

[3] Capgras J., Reboul-Lachaux J. (1923). L’illusion Des Socies Dans Undelire Systematise Chronique. *Bulletin de la Société Clinique de Médecine Mentale*.

[4] Courbon P., Fail G. (1927). Illusion De Fregoli. *Bulletin de la Société Clinique de Médecine Mentale*.

[5] Courbon P., Tusques J. (1932). Illusions D’intermetamorfose et de la Charme. *Annales Médico-psychologiques, Revue Psychiatrique*.

[6] Christodoulou G. N. (1978). Syndrome of Subjective Doubles. *American Journal of Psychiatry*.

[7] Silva J. A., Leong G. B., Shaner A. L., Chang C. Y. (1989). Syndrome of Intermetamorphosis: A New Perspective. *Comprehensive Psychiatry*.

[8] Leis K., Mazur E., Racinowski M. (2019). Delusional Misidentification Syndrome: Dissociation Between Recognition and Identification Processes. *Acta Neuropsychologica*.

[9] Klein C. A., Hirachan S. (2014). The Masks of Identities: Who’s Who? Delusional Misidentification Syndromes. *The Journal of the American Academy of Psychiatry and the Law*.

[10] Grover S., Yadav N. (2025). Misidentification Syndromes: A Retrospective Study From India. *Asian Journal of Psychiatry*.

[11] Manjunatha N., Sarma P. K., Math S. B., Chaturvedi S. K. (2010). Delusional Procreation Syndrome: A Psychopathology in Procreation of Human Beings. *Asian Journal of Psychiatry*.

[12] Silva A., Leong G. B., Shaner A. L. (1991). The Syndrome of Intermetamorphosis. *Psychopathology*.

[13] Fish, Patricia C., Brendan K. (2019). *Fish’s Clinical Psychopathology*.

